# Single-cell biology to decode the immune cellular composition of kidney inflammation

**DOI:** 10.1007/s00441-021-03483-y

**Published:** 2021-06-14

**Authors:** Yu Zhao, Ulf Panzer, Stefan Bonn, Christian F. Krebs

**Affiliations:** 1 Hamburg Center for Translational Immunology (HCTI), University Medical Center Hamburg-Eppendorf, Hamburg, Germany; 2 Institute of Medical Systems Biology, University Medical Center Hamburg-Eppendorf, Hamburg, Germany; 3 Center for Biomedical AI, University Medical Center Hamburg-Eppendorf, Hamburg, Germany; 4 Translational Immunology, III. Department of Medicine, University Medical Center Hamburg-Eppendorf, Hamburg, Germany

**Keywords:** Single-cell biology, Single-cell RNA profiling, Renal function

## Abstract

Single-cell biology is transforming the ability of researchers to understand cellular signaling and identity across medical and biological disciplines. Especially for immune-mediated diseases, a single-cell look at immune cell subtypes, signaling, and activity might yield fundamental insights into the disease etiology, mechanisms, and potential therapeutic interventions. In this review, we highlight recent advances in the field of single-cell RNA profiling and their application to understand renal function in health and disease. With a focus on the immune system, in particular on T cells, we propose some key directions of understanding renal inflammation using single-cell approaches. We detail the benefits and shortcomings of the various technological approaches outlined and give advice on potential pitfalls and challenges in experimental setup and computational analysis. Finally, we conclude with a brief outlook into a promising future for single-cell technologies to elucidate kidney function.

## Introduction

The immune system is a complex network composed of various cell types that interact with each other and with parenchymal cells in the tissue. Its function or dysfunction is pronounced in inflammatory diseases, where various immune cells can play a central role in disease pathogenesis. It is the cross-talk between many types of cells that in fact mediates immune processes (Hewitt and Lloyd 2021). Depending on the specific micro-environmental context, the immune cells communicate with each other and with parenchymal cells of the particular organ (Masopust and Soerens 2019). Characterizing the participating cell types, their cellular networks, unique pathways, genes, and interactions might be key to understand the immune-pathogenesis of immune-mediated kidney diseases. This can provide the basis for manipulating the immune system in a targeted approach.

During the past 20 years, sequencing technologies have revealed a detailed picture of the human genome (McGuire et al. 2020). The roles of genes and transcripts in the development of organisms and disease have been intensively investigated (Rahman et al. 2020). Genome-wide transcriptional profiling paves the way for comprehensive measurements of the molecular state of cells, in lieu of strategies based on selected markers (Yofe et al. 2020). Up until recently, comprehensive genomic analyses relied either on pooling heterogeneous mixtures of cells or on sorting and then profiling subpopulations (Kulkarni et al. 2019). While bulk profiling can provide expression averages, which enables the identification of group differences between a healthy state and disease, it cannot differentiate between changes in cell proportions and cell type-specific gene expression changes (Papalexi and Satija 2018). While recent approaches in cell deconvolution algorithms allow for approximate estimation of cell proportions from bulk profiling data, they still lack in accuracy and cell type-specific gene expression determination (Menden et al. 2020).

In the past 5 years, technical progress has enabled the high-throughput analysis of single cells. It is now possible to simultaneously measure thousands of genes and transcripts across thousands of individual cells using microfluidic approaches (Papalexi and Satija 2018). This is made possible by trapping single cells in water droplets in an oil phase. Recent technical advances allowed for single-cell RNA sequencing (scRNA-seq) of very small samples, enabling the profiling of e.g., human biopsies in clinical settings (Braga et al. 2019; Haber et al. 2017; Krebs et al. 2020a; Zheng et al. 2017). In contrast to FACS-sorting and plate-based scRNA-seq techniques, microdroplet-based scRNA-seq approaches cannot directly link protein and transcriptional expression information. Since cell surface proteins are a common means to define cell types and RNA for these proteins might be lacking, purely scRNA-seq-based cell type detection is often difficult. To link the surface protein and transcription profile at the single-cell level for the microdroplet-based approaches, CITE-seq (Stoeckius et al. 2017) was developed and has been widely used in the immune single-cell studies.

B cells and T cells are the adaptive arm of the immune system, and B cells produce antibodies that can neutralize or opsonize pathogens. These antibodies are also present on the cell surface known as B cell receptor (BCR). T cells are defined by the T cell receptor (TCR), which mediates recognition of pathogen- associated epitopes through interactions with peptide and major histocompatibility complexes (pMHCs) (Peters et al. 2020). BCRs/TCRs are generated by genomic rearrangement of the germline BCR/TCR locus, a process termed V(D)J recombination, that has the potential to generate marked diversity of BCRs/TCRs (estimated to exceed 10^15^ possible receptors) (De Simone et al. 2018). Using paired B/T cell receptor sequencing to study V(D)J recombination at the single-cell level (Stubbington et al. 2016) enables researchers to assess BCR/TCR-based clonality and cell migration, while providing deep insights into cellular function and activation.

In this review, we highlight recent advances in scRNA-seq technology and their application to elucidate kidney function in health and disease, with a special focus on immune cells. Combining single-cell transcriptome, BCR/TCR, and CITE-seq information will provide deep mechanistic insights into kidney inflammation and highlight potential novel cell type and organ-specific therapeutic avenues.

## Single-cell expression profiling basics

The earliest scRNA-seq study was conducted in 2009 by Tang et al. (2009), in which the transcriptome of a single-cell was analyzed. After around 5 years of technical improvement, the throughput and quality of the scRNA-seq was dramatically improved. Current standard scRNA-seq protocols include tissue dissociation, single-cell isolation, cell lysis, and reverse transcription, followed by PCR amplification and sequencing (Wu and Humphreys 2017). The key method of isolating single cells is the introduction of the cellular barcoding technique (Stewart et al. 2020). Cells are sorted into multi-well plates or captured in nanoliter droplets. In each small compartment, the single cell will be lysed and the mRNAs from this particular cell will be labeled with a unique cell barcode during the cDNA synthesis. After pooling of the cDNA, the cell barcodes can later be used to trace back the cell origin of each mRNA transcript (Macosko et al. 2015). Detailed comparisons of plate and droplet-based scRNA-seq methods have been reviewed before (Papalexi and Satija 2018; Potter 2018). In brief, plate-based approaches can capture full-length mRNAs and usually capture more transcripts per cell but suffer from relatively low throughput and higher cost (Papalexi and Satija 2018). On the contrary, droplet-based methods are more cost efficient and allow high throughput of up to millions of cells but do not sequence the full length of the transcript.

The choice of sequencing platform has actual ramification for the subsequent information obtained. If deep molecular and splicing information per cell is of essence, then maybe, a plate-based assay should be taken. If it is important to capture many cells and maybe highlight sparse cellular subpopulations with a limited budget, droplet-based assays are favorable.

## Single-cell transcriptome atlases of the kidney in health and disease

**Fig. 1:**
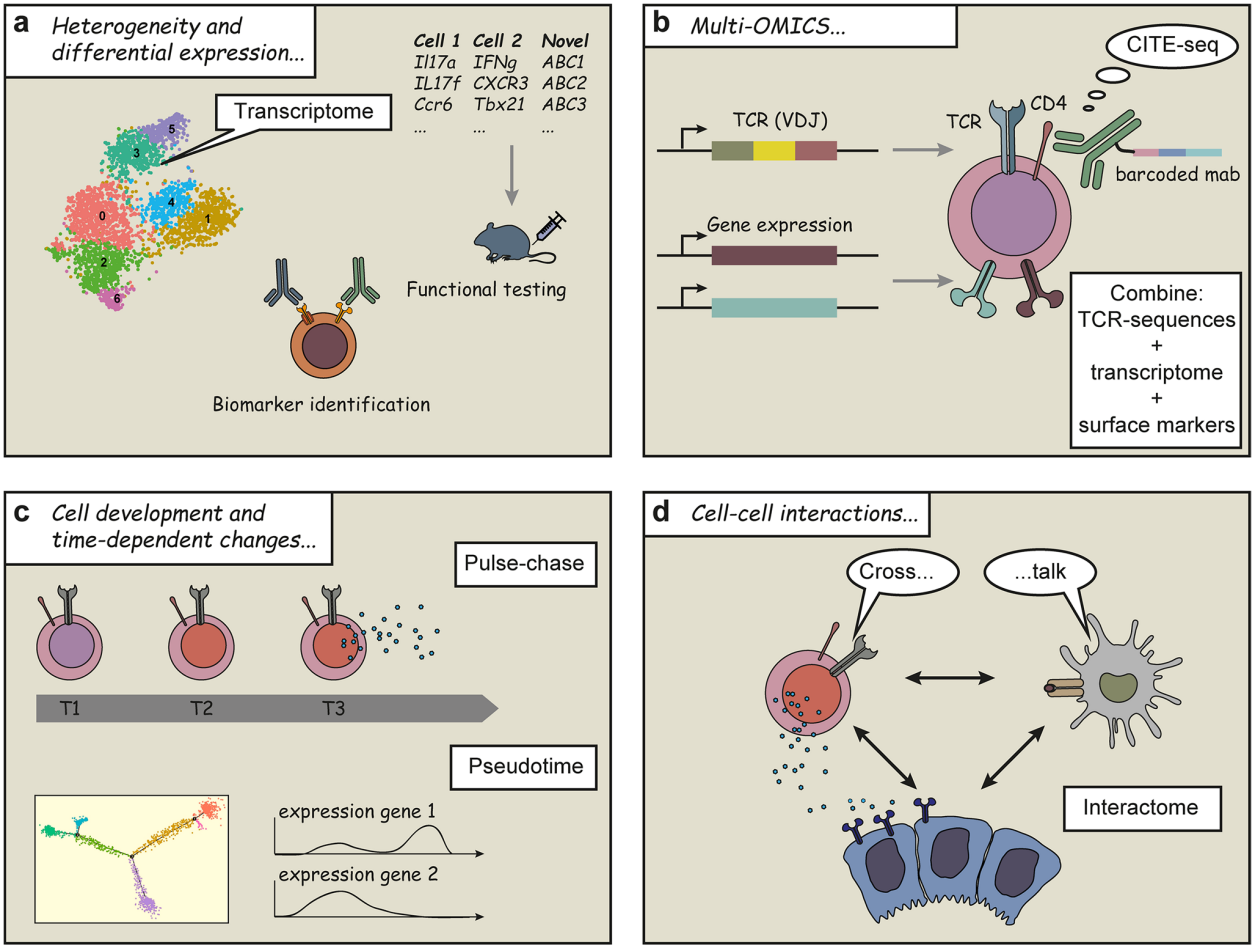
Different approaches to high-dimensional analysis of cells by single-cell techniques. This figure gives an overview of some of the many possible applications of single-cell expression profiling. The heterogeneity of cells can be uncovered by gene expression analysis at the single-cell level. This can result in the identification of new biomarkers or in the generation of new hypothesis that can be tested for example in animal models (**a**). Multi-OMIC approaches can be performed by combining gene expression analysis with genetic modifications (T or B cell receptor rearrangement) and protein identification in individual cells (**b**). Developmental trajectories can be investigated by pseudotime analysis (**c**). Cell–cell interactions can be scrutinized by identifying ligand and receptor matches on different cells (**d**)

Single-cell technologies can be employed to uncover the cellular heterogeneity of cells within the kidney (Fig. 1). One of the first single-cell atlases of mouse kidney was reported in 2018 by Park and colleagues (Park et al. 2018). They performed scRNA-seq of 57,979 murine kidney cells and identified 21 major tubular and glomerular cell types. Besides the cell type identification, they also addressed the cell type specificity of the kidney disease GWAS genes (genome-wide association study) using the expression matrix. Other studies that profiled human kidney single-cell expression were published around the same time (Liao et al. 2020; Sivakamasundari et al. 2017; Wilson and Humphreys 2019). In general, human and murine kidney cell compositions are quite similar in healthy individuals, while human kidney biopsies from allografts (Wu et al. 2018), tumors (Young et al. 2018), and other kidney diseases such as diabetic kidney disease (Wilson et al. 2019), IgA nephropathy (Zheng et al. 2020), and lupus nephritis (LN) showed differences to healthy murine kidney (Der et al. 2019). These studies provided first insights into renal cell heterogeneity and cell type-specific responses to disease. However, in these first kidney cell atlases, tubular and endothelial cells constituted the vast majority of the observed cell types, while rare cell types, such as immune cells, were hardly detected in healthy and diseased human and murine samples. To fully grasp the impact of the immune cells in renal pathology, a detailed kidney immune landscape would be essential.

## Immune landscape of the diseased human kidney

A recent study explored kidney immune cell heterogeneity in lupus nephritis (LN) patient biopsies (Arazi et al. 2019). The researchers sorted leukocytes with flow cytometry and then performed scRNA-seq. After clustering analysis, they identified 5 macrophage clusters, 7 T cell clusters (including NK cells), and 4 B cell clusters. After normalization and comparison with one living donor control, they showed that the type I interferon response score is higher in LN patients. The IFN signaling pathways were suggested to be potential prognostic markers of LN.

Another interesting recent publication by Stewart et al. profiled the positioning of cells in the kidney (Stewart et al. 2019). By investigating cells from the human kidney and using machine learning to reconstruct the spatial information, they inferred gross anatomical positioning of a cell in the renal tissue. This approach has been summarized and discussed in more detail recently (Krebs et al. 2020b).

## Using CITE-seq to investigate renal T cell subtypes

Approaches that solely rely on profiling single-cell transcripts yield good results when used for the quantification and characterization of cell types that can be profiled in sufficient numbers, e.g., T cell, B cell, and myeloid cell types. For the detection and clustering of rare subpopulations of immune cells, however, the molecular information obtained usually does not suffice to reliably classify these cell types. This problem can be solved by increasing the cell number, which easily gets prohibitively expensive, by presorting of specific cell types using magnetic-of FACS-sorting strategies, or by using CITE-seq antibody-based labeling of cells (Stoeckius et al. 2017).

Three potential challenges of T cell subtype investigation using scRNA-seq, only, are as follows: First, not all the surface markers which define T cell subpopulation have high mRNA expression. In our in-house human renal biopsy scRNA-seq datasets, we noticed that CD4 mRNA expression is low, compared to the detection of surface CD4 protein in e.g., FACS analysis (Krebs et al. 2020c). This observation is in line with publications from other research groups (Ding et al. 2020; Zemmour et al. 2020). Second, some surface protein markers share the same gene origin and are the products of alternative splicing. The most relevant example might be the gene *PTPRC*, which encodes for CD45, CD45RA, and CD45RO. CD45 is expressed on all leukocytes and CD45RA is a marker for naïve T cells whereas CD45RO marks memory T cells (Masopust and Soerens 2019). The expression of *PTPRC* obtained by scRNA-seq approaches cannot easily distinguish the CD45RA and CD45RO T cells if not full-length techniques are employed (Ntranos et al. 2019). Third, single-cell dissociation and other experimental procedures might induce stress and other responses in select cells, resulting in gene expression that might obscure cell identity (O’Sullivan et al. 2019). CD69 protein is expressed in most of the tissue resident memory T cells (Trm cells), but it is also a marker of early activation (Kumar et al. 2017). In our in-house human renal biopsy scRNA-seq datasets, we detected CD69 mRNA almost in all the T cells, although the flow cytometry data shows that only a subset of cells is CD69^+^.

These three potential challenges can be resolved by simultaneous measurement of scRNA-seq and surface proteins, a technique named CITE-seq (cellular indexing of transcriptomes and epitopes by sequencing) (Stoeckius et al. 2017). The core idea of CITE-seq is conjugating polyadenylated DNA barcodes to antibodies targeting cell surface proteins so that the surface proteins can be translated into sequenceable information. The DNA barcode can be captured together with the mRNA from target cells. After the reverse transcription step, a separate cell surface protein-specific library can be obtained and sequenced. The same cell barcode for both RNA and protein antibody will later enable overlap of CITE-seq and scRNA-seq data. Compared to the flow cytometry technique, the advantage of CITE-seq is that it can measure tens of proteins, while the number of fluorescence labels for antibodies in flow cytometry is much more limited.

We performed CITE-seq and scRNA-seq together in our antineutrophil cytoplasmic antibody (ANCA)–associated glomerulonephritis (GN) patients’ renal biopsy T cells (Krebs et al. 2020c). Indeed, our CITE-seq data showed robust signal of CD4 surface proteins and could easily distinguish CD45, CD45RA, and CD45 RO. The CD69 protein expression is more restricted to a subgroup of T cells and we identified CD69^+^/CCR6^+^ Trm17 cells in the datasets from the kidney. We also further validated the tissue resident signature of the Trm clusters by overlapping them with previous reported human Trm signatures (Kumar et al. 2017). By performing functional experiments in animal models, we have identified a T cell subset (Trm17 cells) that can be induced by bacterial infections and reactivated in unrelated inflammation to produce IL-17A and contribute to tissue damage.

## Single-cell VDJ-seq to understand clonal expansion in the kidney and across tissues

In viral or bacterial infections, the antigen-specific naïve B/T cells can divide and expand themselves profoundly (Tu et al. 2019). B/T cell infiltration and expansion have been linked to multiple autoimmune diseases such as multiple sclerosis (MS) (Pappalardo et al. 2020; Arneth 2019), inflammatory bowel disease (Smillie et al. 2019; Mizoguchi et al. 2017), and glomerulonephritis (Krebs et al. 2017; Schrezenmeier et al. 2018), while it is still unclear whether auto-antigen triggered B/T cell expansion occurs in immune-medicated kidney disease (Kitching et al. 2020).

To understand the expansion of B/T cell antigen receptor (BCR/TCR)–specific cell clones, it is essential to sequence variable regions of the BCRs/TCRs that confer antigen specificity, as well as the gene expression of the corresponding cells, a technique called single-cell BCR/TCR sequencing (O’Sullivan et al. 2019). BCR/TCR sequencing can also serve as a natural barcode to trace B/T cell migration between the kidney, lymph nodes, and peripheral blood.

In our very recent study of severe COVID-19 patients (Zhao et al. 2021), we identified clonally expanded tissue-resident memory-like Th17 cells (Trm17 cells) in the lungs by single-cell sequencing of TCRs and RNA from sorted T cells. In fact, this is the first use of this technique to trace the T cell clones across tissues in these patients. Our analysis further shows these clonally expanded Trm17 cells express high levels of cytokines such as GM-CSF and IL-17A, molecules both implicated in cytokine storms observed in patients with severe COVID-19.

To our knowledge, similar studies about single-cell BCR/TCR sequencing in the autoimmune kidney diseases have not been reported yet, while it is tempting to speculate that renal resident B/T cells might expand upon stimulation and contribute to the pathogenic process in kidney inflammation. Another unanswered question is whether the origin of inflammation resides in the kidney or other organs. In particular in systemic vasculitis like ANCA-assosciated GN (Kitching et al. 2020), tracking the clones across tissues might be a potential way to shed light on the cellular origin and relations in kidney inflammation.

## Immune cell interactome

**Fig. 2:**
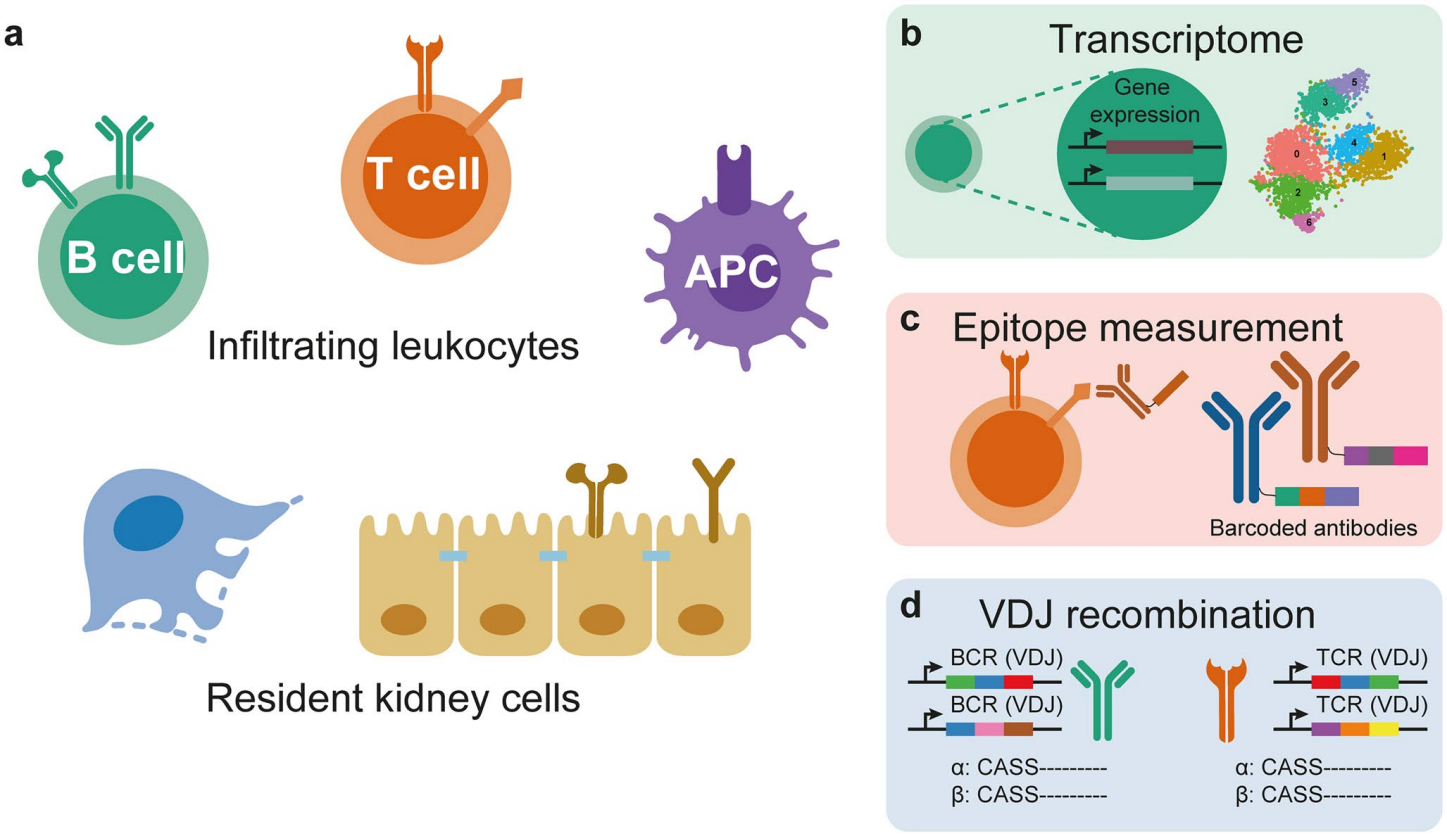
The combination of single-cell transcriptome sequencing with surface protein measurement and VDJ sequencing. The renal tissue is composed of resident kidney cells, including epithelial cells (podocytes, tubular epithelial cells), infiltrating leukocytes (such as B cells, T cells, and myeloid cells), and others (**a**). Single-cell technologies can be used to combine transcriptome sequencing (**b**), epitope measurement of cell surface molecules (**c**), and V(D)J recombination of the T and B cell receptors (**d**)

Investigating different roles of each immune cell population is crucial to decode the inflammation; however, the immune cells also interact with each other and with parenchymal cells of the kidney via chemokines, cytokines, and their respective receptors (Fig. 1). Using known receptor–ligand interactions, scRNA-seq data can be used to computationally derive potential cellular crosstalk (Arazi et al. 2019; Stewart et al. 2019; Wu et al. 2018). For example, in the LN study by Arazi et al., the authors analyzed the chemokine- and cytokine-mediated cellular networks between the characterized immune cell types and suggested CXCR4 and CX3CR1 as potential therapeutic targets. A more convenient computational tool and database, called CellPhoneDB, has been made to explore ligand–receptor interactions using single-cell data (Efremova et al. 2020). In our study of patients with COVID-19, CellPhoneDB enabled us to obtain a detailed interactome of lung immune cells. Our cell cross-talk analysis suggested that Trm17 cells could potentially interact with other cells associated with COVID-19 severity and lung damage, such as lung macrophages and CD8^+^ killer T cells (Zhao et al. 2021). In a kidney setting, computational analysis using CellPhoneDB or similar information could be used to understand the interaction of podocytes and T cells, for example (Fig. 2).

## Key challenges in kidney single-cell immunology

### Sample availability and batch effects

The investigation of immune and tissue cell signaling in solid tissues is complicated by the necessary dissociation of the sample prior to scRNA-seq. The acquisition of fresh samples, the dissociation of the sample into single cells, and the timely further extraction and processing of the RNA for scRNA-seq pose significant challenges and room for error. Over-digestion of samples to extract single cells, for example, will result in stressed and dead cells and bad quality scRNA-seq data. The cell number, isolation efficiency, and experimental bias might all influence the downstream analysis (Saelens et al. 2019). Lots of efforts have been made by the bioinformatics community to remove the batch effects and integrate the different datasets (Stuart et al. 2019). The major challenge in the batch correction field is to remove the technical bias while maintaining the biological differences between samples. Over-correction of samples will hamper the biological interpretation of datasets. The major batch correction methods have evolved from Bayesian algorithms, such as Limma (Smyth and Speed 2003) and ComBat (Johnson et al. 2007), to PCA (principal component analysis), CCA (canonical correlation analysis), MNN (mutual nearest neighbors), and deep learning-based approaches, such as Harmony (Korsunsky et al. 2019), Seurat v2 (Butler et al. 2018), Scanorama (Hie et al. 2019), Seurat v3 (Stuart et al. 2019), LIGER (Welch et al. 2019), and BERMUDA (Wang et al. 2019). Detailed comparisons between multiple batch correction approaches have been summarized recently (Oller-Moreno et al. 2021; Li et al. 2020; Tran et al. 2020). For the kidney immune single-cell datasets, especially the patient biopsy datasets, suitable batch correction methods need to be evaluated within the community in the future.

### Cell subtype identification

Cell type identification is the most crucial step after data quality control and integration. Many downstream interpretation steps rely on the accurate classification of cell types. Determining cell types for individual cells is currently very difficult due to noise and technical zeros (dropouts) in single-cell RNA sequencing. As a result, most of the current cell type identification approaches use common expression patterns of marker genes to identify cell types (Oller-Moreno et al. 2021). The common unsupervised clustering algorithms include partitioning, hierarchical clustering, or graph-based clustering (Petegrosso et al. 2020; Zheng and Wang 2019). Meanwhile, with the increasing of more annotated single-cell datasets, computational biologists also developed supervised methods based on machine learning or deep learning, such as scPred (Alquicira-Hernandez et al. 2019), MARS (Brbić et al. 2020), and rCASC (Alessandrì et al. 2019). The amount of single-cell datasets containing detailed characterization of immune cells from the kidney is still limited. Therefore, we need more efforts in the future to apply the supervised algorithms to identify the immune cell subtypes in renal inflammatory diseases.

### Temporal dynamics

All the biological events in the body are complicated dynamic processes such as T cell polarization and activation upon antigen stimuli. However, current single-cell methods are all snapshots of these dynamic processes. It is technically difficult to obtain human renal tissues at different time points and if possible, there is bias given by a different piece of tissue. The computational biologists developed multiple pseudotime analysis algorithms such as PAGA (Wolf et al. 2019), Monocle (Trapnell et al. 2014), Slingshot (Street et al. 2018), and single-cell RNA velocity (Bergen et al. 2020) to infer the continuous processes. The processes are reconstructed by finding paths through cellular space that minimize transcriptional changes between neighboring cells. The performance of different pseudotime algorithms can be very variable across datasets. A detailed benchmarking on those pseudotime methods has been performed by Saelens et al. (2019). Since the immune process in the tissue is not clearly studied, how much of the temporary dynamics can be reflected through the transcriptional similarity is still an uncertain question. This should also be addressed systematically in different experimental animal models.

### Spatial organization

While the single-cell sequencing techniques described so far capture molecular profiles of single cells at unprecedented depth, they usually do not confer spatial information of where in the tissue the single cells originate from or which cells they interacted with. This information, however, is critically relevant in the kidney and other organs, as different kidney compartments have variable micro-environment conditions such as sodium and oxygen concentration gradients in the cortex and medulla (Stewart et al. 2020). It is therefore quite likely that immune and tissue cells in different macro- and micro-environments expose different gene regulation, signaling, and activity states. Unsurprisingly, most of the current single-cell kidney data do not capture spatial information, while some recent studies provide insights into the immune topology of the human kidney (Stewart et al. 2019). The recent development of single-cell spatial transcriptomic technologies, such as MERFISH (multiplexed error-robust fluorescence in situ hybridization) (Moffitt et al. 2018) and STARmap (spatially resolved transcript amplicon readout mapping) (Wang et al. 2018), paves the way to spatial single-cell transcriptomic experiments on renal tissues, to leverage information contained in local cell interactions (Andersson et al. 2020). How to apply these methods to different kidney disease samples will also be of major importance to dissect the renal immune spatial organization.

### Epigenetic landscape of the genome

While the gene expression program is quick to respond to external and internal stimuli per se, epigenetic changes of the chromatin can restrict, expand, or change the repertoire of expression changes a cell can make. Especially in the context of mid- to long-term activation and signaling changes of cell types, such as tissue resident memory cells in the kidney, it is important to profile and understand underlying epigenetic changes. Two methods of choice that allow for single-cell epigenetic profiling are scATAC-seq (single-cell assay for transposase-accessible chromatin using sequencing) (Buenrostro et al. 2015) and scChIP-seq (single-cell chromatin immunoprecipitation followed by sequencing) (Rotem et al. 2015). Especially scATAC-seq is a cost-effective and rather reliable technology for single-cell epigenetic profiling, as exemplified in a scATAC-seq study on murine kidney (Cusanovich et al. 2018). While studies on single-cell epigenetic regulation (of immune cells) in healthy or diseased kidneys are still rare, we expect to see a surge in studies investigating epigenetic mechanisms of gene regulation in conjunction with scRNA-seq data soon.

## Concluding remarks

Single-cell genomics serves as a molecular microscope for the observation of cellular landscapes of different tissues and cell types (Giladi and Amit 2017). The first kidney single-cell atlases have been established by the latest advancements in the field of scRNA-seq and analysis of many millions of cells from renal tissue. Combining simultaneous epitope measurement with gene expression data gives additional power to identify subtypes and states of immune cells. The VDJ-seq technology will provide further insights into lymphocytic clonal expansion and lineages. Ligand–receptor analysis based on single-cell data reveals the interactome across cell populations. We listed several major challenges in the renal immunology research. In the future, we expect to observe spatial single-cell sequencing technology applied to kidney research, potentially augmented by temporal information via multi-label (life) imaging (Zimmermann et al. 2021). Spatial and clonal information might pave the way to understand localized immune action in its tissue context to grasp mechanisms of renal inflammation and injury. We believe that single-cell techniques will become routine methods to elucidate the mechanisms that underlie kidney inflammation, paving the way for novel treatment strategies.
